# Plin5/p-Plin5 Guards Diabetic CMECs by Regulating FFAs Metabolism Bidirectionally

**DOI:** 10.1155/2019/8690746

**Published:** 2019-10-17

**Authors:** Jin Du, Juanni Hou, Juan Feng, Hong Zhou, Heng Zhao, Dachun Yang, De Li, Yongjian Yang, Haifeng Pei

**Affiliations:** ^1^Department of Cardiology, The General Hospital of Western Theater Command, Chengdu 610083, China; ^2^Department of Gastroenterology, The General Hospital of Western Theater Command, Chengdu 610083, China; ^3^Department of Respiration, The General Hospital of Western Theater Command, Chengdu 610083, China; ^4^Department of Function, Sichuan Petroleum General Hospital, Chengdu 610212, China

## Abstract

**Background:**

Hyper-free fatty acidemia (HFFA) impairs cardiac capillaries, as well as type 2 diabetes mellitus (T2DM). Perilipin 5 (Plin5) maintains metabolic balance of free fatty acids (FFAs) in high oxidative tissues via the states of nonphosphorylation and phosphorylation. However, when facing to T2DM-HFFA, Plin5's role in cardiac microvascular endothelial cells (CMECs) is not defined.

**Methods:**

In mice of WT or Plin5^−/−^, T2DM models were rendered by high-fat diet combined with intraperitoneal injection of streptozocin. CMECs isolated from left ventricles were incubated with high glucose (HG) and high FFAs (HFFAs). Plin5 phosphorylation was stimulated by isoproterenol. Plin5 expression was knocked down by small interfering RNA (siRNA). We determined cardiac function by small animal ultrasound, apoptotic rate by flow cytometry, microvessel quantity by immunohistochemistry, microvascular integrity by scanning electron microscopy, intracellular FFAs by spectrophotometry, lipid droplets (LDs) by Nile red staining, mRNAs by quantitative real-time polymerase chain reaction, proteins by western blots, nitric oxide (NO) and reactive oxygen species (ROS) by fluorescent dye staining and enzyme-linked immunosorbent assay kits.

**Results:**

In CMECs, HFFAs aggravated cell injury induced by HG and activated Plin5 expression. In mice with T2DM-HFFA, Plin5 deficiency reduced number of cardiac capillaries, worsened structural incompleteness, and enhanced diastolic dysfunction. Moreover, in CMECs treated with HG-HFFAs, both ablation and phosphorylation of Plin5 reduced LDs content, increased intracellular FFAs, stimulated mitochondrial *β*-oxidation, added ROS generation, and reduced the expression and activity of endothelial nitric oxide synthase (eNOS), eventually leading to increased apoptotic rate and decreased NO content, all of which were reversed by N-acetyl-L-cysteine.

**Conclusion:**

Plin5 preserves lipid balance and cell survival in diabetic CMECs by regulating FFAs metabolism bidirectionally via the states of nonphosphorylation and phosphorylation.

## 1. Introduction

The main features of type 2 diabetes mellitus (T2DM) are hyperglycemia and metabolic disturbance of lipids and proteins [1, 2]. More than 67.1% of T2DM patients have abnormal lipid metabolism [3], such as hyper-free fatty acidemia (HFFA) and hypercholesterolemia. Free fatty acids (FFAs), known as nonesterified fatty acids (NEFAs), are closely related to metabolic syndrome in T2DM patients [4] and serve as an important risk factor in cardiovascular diseases [5]. Moreover, within the body, the metabolic processes of glucose and lipid can interact with each other. Lee et al. even have found that plasma FFAs begin to rise progressively as earlier as two weeks prior to hyperglycemia [6]. It is clear that hyperglycemia aggrandizes the vulnerability of endothelial cells to exogenous stimulus, which can be aggravated by hyperlipidemia. Thus, we cannot ignore the interaction of glucose and lipid in research of diabetic complications. It is well known that microvascular lesions widely occurred in T2DM, resulting in abnormal microcirculation [7, 8]. Microvascular endothelial cells form the basic structure of microvessels, and the pathological changes in cardiac microvascular endothelial cells (CMECs) occur before the myocardium in the diabetic heart [9]. Recently, coronary microvascular diseases (CMD) have attracted wide attention, such as cardiac X syndrome, coronary slow flow phenomenon, and no-reflow phenomenon. By using coronary angiography, it is believed that microvascular endothelial dysfunction is an important cause of CMD [10]. Therefore, it is an urgent task to explore the molecular mechanism of CMECs injury, which may help develop new target for CMD.

Lipid droplets (LDs) serve as essential organelles for intracellular lipid storage [11]. Perilipin 5 (Plin5) is one of the important members of lipid droplet-associated proteins on the surface of LDs and is highly expressed in high fatty acid oxidation tissues, such as heart, skeletal muscle, liver, and brown fat [12]. Plin5 plays an important role in cellular lipid metabolism by promoting or inhibiting the hydrolysis of neutral fat in LDs. It not only ensures the energy supply of cells but also prevents the lipid toxicity damage caused by fatty acid overload [13]. A large amount of literatures reveal that Plin5 knockout reduces the formation of LDs, enhances the oxidation of FFAs, and causes insulin resistance [14–16]. In vascular endothelial cells, LDs can be quickly formed and decomposed [17]. Plin5 deletion in large arteries of mice can accelerate the progression of atherosclerosis [18]. On the contrary, it is reported that Plin5 overexpression with high cardiac specificity results in myocardial steatosis and mitochondrial dysfunction in mice [19, 20]. However, the concrete role of Plin5 in CMECs has not been well defined yet. More interestingly, the phosphorylation mode of Plin5 (p-Plin5) can promote the hydrolysis of triglyceride (TG) in LDs and enhance the release of FFAs into the cytoplasm [21]. Many studies have shown that protein kinase A (PKA) activation can stimulate the phosphorylation of Plin5 [22, 23], in which serine 155 may be the important site [22, 24]. Moreover, p-Plin5 in the nucleus can also inhibit SIRT1 activity, promote the transcription of peroxisome proliferator-activated receptor gamma coactivator 1-*α* (PGC-1*α*), and eventually enhance the synthesis and oxidation function of mitochondria [24]. Furthermore, it is reported that increasing the PKA-mediated phosphorylation of Plin5 by atorvastatin can reduce cellular lipid accumulation in hepatocytes [25]. However, the specific action of p-Plin5 in diabetic CMECs remains unclear.

Oxidative stress is caused by excessive production of reactive oxygen species (ROS) and reactive nitrogen (RNS) and/or the reduction of cellular antioxidant capacity [26, 27]. Mitochondria is the most important source of ROS in cells [28], and the *β*-oxidation of FFAs can produce a large number of ROS [29, 30]. Many studies show that Plin5 is closely related to oxidative stress, and Plin5 knockout can lead to enhanced mitochondrial *β*-oxidation in myocardium and hepatocytes in mice [16, 31]. However, whether oxidative stress mediates the biological function of Plin5/p-Plin5 in diabetic CMECs deserves a thorough study.

The aims of this study are to determine (1) whether Plin5 is disturbed in diabetic CMECs, (2) whether the deficiency and phosphorylation of Plin5 affect the survival of CMECs under the condition of T2DM-HFFA, and, if so, (3) to identify whether oxidative stress participates in those biological effects of Plin5/p-Plin5 in CMECs.

## 2. Material and Methods

### 2.1. Preparation of Mice

Normal mice of wild type (WT; C57BL6/J, male) were obtained from Vital River (Beijing, China) and Plin5^−/−^ mice from Graduate School of Life Science (Hyogo, Japan) for this experiment [14]. The reverse transcription-polymerase chain reaction (RT-PCR) was used to detect the genotypes of mice. Mice were given free access to water and feed and raised under standard conditions at 22-26°C with 12-hour light/12-hour dark cycle. These two genotypes mice were both randomly divided into the control group and type 2 diabetes mellitus with hyper-free fatty acidemia (T2DM-HFFA) group. To prepare animal models of T2DM-HFFA, 6-week-old mice were given high-fat diet (60% from fat, 20% from carbohydrates, and 20% from protein, Research Diets, USA) for 8 weeks and then were fasted for 16 hours and subsequently given intraperitoneal injection of streptozocin (STZ; 30 mg/kg, Sigma, USA). Later, these mice were fed continuously with high-fat diet for 16 weeks [32]. The fasting blood glucose levels ≥11.1 mmol/L and with symptoms of polyuria and polydipsia were confirmed to be successful in modeling [33]. Meanwhile, serum FFAs level ≥3.0 mmol/L was defined HFFA [34]. 24 weeks later, mice with hyperglycemia and HFFA were used for the experiments. All experiments were conducted in adherence with National Institutes of Health Guidelines on the Use of Laboratory Animals and were approved by the Institutional Animal Care and Use Committee in the General Hospital of Western Theater Command.

### 2.2. Biochemical Indicator

The mice were given 12 hours of fasting treatment before experiments. Electronic balance was used to detect body weight of mice. Serum levels of glucose, insulin (INS), free fatty acids (FFAs), triglyceride (TG), and cholesterol (CHOL) were determined by automatic biochemical analyzer (Model 7020, Hitachi, Japan).

### 2.3. Scanning Electron Microscope

Mice were anesthetized with 4% chloral hydrate, and the abdominal aorta was intubated. Heparin saline and acetone butanone mixture was perfused in turn. Then, acrylonitrile butadiene styrene (ABS; 10%) solution was perfused into for 15 mL when perfusion pressure was maintained at about 200 mmHg. After the perfusion was completed, the hearts of mice were placed at room temperature for 24 hours and then corroded by concentrated hydrochloric acid for 1 week. Microvascular casts were obtained by rinsing with water and dried by freeze-drying. After spraying with gold, the microvasculatures were observed by the scanning electron microscope (Model S-3400N, Hitachi, Japan) at 15 kV.

### 2.4. Immunohistochemical Staining

After fixed with formaldehyde, the heart was given treatment of paraffin embedding and finally made into slices with a thickness of 5 *μ*m. Sections were incubated with hydrogen peroxide (H_2_O_2_; 3%, 25 minutes) to eliminate the endogenous peroxidase. Then, sections were covered with bovine serum albumin (BSA; 3%, 30 minutes) at room temperature. After the rabbit anti-mouse CD31 primary antibody (1 : 200, CST, USA) was added, sections were kept in the wet box (4°C, overnight). After washed with phosphate-buffered saline (PBS), goat anti-rabbit secondary antibody (1 : 1000, CST, USA) was added and incubated at 37°C for 2 hours. The sections were observed under an optical microscope (DM3000, Leica, Germany). The microvessels were the standard of blood vessel with diameter less than 100 *μ*m. Each slice was randomly observed for 5 visual fields, and the mean numbers of microvessels were calculated with Image-Pro Plus.

### 2.5. Cardiac Function

The mice were anesthetized with 4% chloral hydrate. VEVO 2100 high-resolution in vivo imaging system (Visual Sonics, Canada) was used to measure the left ventricular early mitral diastolic wave/late mitral diastolic wave (E/A) ratio and the percentage of left ventricular ejection fraction (LVEF). The whole detection process was carried out by an ultrasonic technician who was blinded to the experimental groups, and the detection parameters remained unchanged.

### 2.6. Preparation of CMECs

CMECs were isolated from the hearts as previously described [35, 36]. Briefly, the hearts of experimental mice were washed with precooled PBS at 4°C. After the aorta and atrial tissues were dislodged, the epicardium and endocardium were removed from ventricular tissues which were then cut into slices of 1 mm^3^ size pieces and incubated in collagenase II (0.2%, Sigma, USA) for 30 minutes. After that, 100 *μ*m strainer mesh was used to remove the undigested tissues. Finally, cells were cultured in the Dulbecco's modified Eagle's medium (DMEM; HyClone, USA) supplemented with fetal bovine serum (FBS; 15%, Gibco, USA). The cells for transplantation were purified using differential time adherent method. Once cell confluence reaches 80%, cells were digested with trypsin (0.25%, 37°C, 20 minutes; Sigma, USA). The third to fifth generation cells were harvested for the further experiments. CMECs were positively identified by staining of von Willebrand factor (vWF) and CD31. Media were replaced to different conditions 24 hours after cell inoculation: normal glucose medium (normal, 5.5 mmol/L), high glucose medium (HG, 25 mmol/L), high free fatty acids medium (HFFAs; 1 mmol/L, Sigma, USA), and high glucose plus high free fatty acids medium (HG-HFFAs). HFFAs were a mixture of palmitate and oleate, 1 : 2 (*w*/*w*) [37]. Then, the cells were incubated for another 24 hours. Reagents and drugs used in cell experiments in vitro were as follows: N-acetyl-L-cysteine (NAC; 10 nmol/L, 8 hours; Sigma, USA) and isoprenaline (ISO; 10 *μ*mol/L, 8 hours; Sigma, USA).

### 2.7. Cellular Immunofluorescence

CMECs were fixed in paraformaldehyde at room temperature for 30 minutes. Triton (0.1%, 5 minutes) was used to broken film. Then, cells were covered with 3% BSA for 30 minutes. Rabbit anti-mouse Plin5 primary antibody (1 : 500, Novus, USA) was added and kept overnight at 4°C. The donkey anti-rabbit fluorescent secondary antibody (1 : 1000, Abcam, USA) was added the next day, and the cells were observed under laser confocal microscopy (LSCM; FV 1000, Olympus, Japan) after cleaning with PBS for 3 times.

### 2.8. Cell Apoptosis

Cell apoptosis was measured by Annexin V-FITC/PI assay kit (BD, USA). After digestion, CMECs were washed 2 times with cooled PBS. Then, cells were suspended with 1× binding buffer and added with Annexin V-FITC and PI 2.5 *μ*L each. CMECs were incubated under light avoidance conditions at room temperature for 15 minutes. Apoptosis was detected by flow cytometry (Partec, Germany), and data were analyzed by FlowJo software.

### 2.9. Nitric Oxide (NO)

When the intervention was completed, CMECs were incubated with DAF-2DA, a kind of fluorescent probe for the detection of NO (5 *μ*mol/L, 20 minutes; Abcam, England). After washed by PBS for 3 times to remove the unbound DAF-2DA, CMECs were observed under laser confocal microscopy (LSCM; FV 1000, Olympus, Japan) at 488 nm emission wavelengths. In addition, the level of NO in CMECs was also detected by enzyme-linked immunosorbent assay (ELISA) kit (Elixir, Canada) following the instruction [38].

### 2.10. Intracellular ROS

According to the 1 : 1000 ratio, dihydroethidium (DHE; Beyotime, China) was diluted with Hank's balanced salt solution (HBSS; Invitrogen, USA) to make the final concentration (5 *μ*mol/L). CMECs were incubated with DHE in the incubator of 37°C for 45 minutes. After washed with HBSS, cells were observed under fluorescence microscope (Olympus, Japan). Besides, the content of intracellular ROS was also detected by ELISA kit (Elixir, Canada) following the instruction [39].

### 2.11. Gene Expression

Total RNA was extracted from cells using TRIzol reagent (Invitrogen, USA), quantitated using a NanoDrop 2000 spectrophotometer. Then, the RNA was reverse-transcribed using a kit (TaKaRa, Japan). Quantitative real-time polymerase chain reaction (qRT-PCR) was performed in a CFX96 real-time detection system (Bio-Rad, USA) using SYBR Green PCR kit (TaKaRa, Japan). Relative mRNA levels were quantified using the comparative *ΔΔ*CT method with GAPDH as reference gene. The following primer sequences were used for qRT-PCR: GAPDH forward, 5′-AGGTCGGTGTGAACGGATTTG-3′, reverse, 5′-TGTAGACCATGTAGTTGAGGTCA-3′; Plin5 forward, 5′-GAAGTGGGCACAGTGGAGG-3′, reverse, 5′-AAAGAGTGTTCATAGGCGAGAT-3′; and carnitine palmitoyl transferase 1 (CPT-1) forward, 5′-CACTGCAGCTCGCACATTAC-3′, reverse, 5′-CCAGCACAAAGTTGCAGGAC-3′.

### 2.12. Western Blot

CMECs were washed with PBS for 3 times; total protein and nuclear protein were extracted by a kit (Sigma, USA). The concentration of protein was determined with the Bio-Rad protein assay kit (Bio-Rad, USA). Equivalent amounts of total protein (25 *μ*g) were subjected to SDS-PAGE and transferred to PVDF membranes. The membranes were incubated with specific primary antibodies against Plin5 (1 : 2000, Novus, USA), GAPDH (1 : 3000, Novus, USA), Histone H3 (1 : 2000, CST, USA), and eNOS (1 : 2000, Novus, USA) for overnight. After incubation with goat anti-rabbit IgG secondary antibody (1 : 2000, 1 hour; Thermo Fisher, USA) at room temperature, proteins were detected with enhanced chemiluminescence and quantified using Image-Pro Plus software [40].

### 2.13. Nile Red Staining

The cells were rinsed 2 times with PBS and fixed by incubating with 2 mL 4% (*w*/*v*) paraformaldehyde at room temperature for 30 minutes. After that, the cells were washed 3 times with PBS. Nile red stock solution (1 mg/mL, 1 *μ*L) was added to 10 mL of PBS. Then, the cells were covered with 1 mL Nile red staining solution and incubated for 10 minutes, all of which were protected from ambient light. The cells were washed with PBS 3 times and observed under laser confocal microscopy. The fluorescence intensity was analyzed by ImageJ software.

### 2.14. Intracellular FFAs

CMECs were homogenized in 200 *μ*L of 1% (*w*/*v*) Triton X-100 in chloroform solution. The samples were centrifuged at 13,000 g for 10 minutes to remove insoluble material. We collected the organic phases (lower phase) and removed chloroform by air dry at 50°C. The dried lipids were dissolved in 200 *μ*L fatty acid assay buffer by vortexing extensively for 5 minutes. Then, the concentration of FFAs was determined by a Free Fatty Acid Quantitation Kit (Sigma, USA), according to the instruction.

### 2.15. siRNA Transfection

One day before transfection, the cells were inoculated into 6-well plates and cultured in antibiotic-free medium. Moderate transfection reagent was added Opti-MEM medium and incubated at room temperature for 5 minutes. Small interfering RNA (siRNA), including the control siRNA and Plin5 siRNA (Invitrogen, USA), was added into Opti-MEM medium and blended. Then, the diluted siRNA was gently mixed with the incubated transfection reagent and incubated for 30 minutes at room temperature. Next, we removed the previous medium from the 6-well plates, and added 1.5 ml new antibiotic-free medium and 500 *μ*l siRNA-transfection reagent in it. The cells were placed in the incubator for 48 hours and then intervened as planned.

### 2.16. Statistical Analyses

Data were shown as means ± standard errors of the means (SEM). The independent sample *t*-test was used in the comparison between the 2 groups. The single-factor *ANOVA* was used in comparison among multiple groups, and pairwise comparison in multiple groups was conducted with LSD *t*-test. *P* ≤ 0.05 was considered significant. All statistical tests were performed using GraphPad Prism software version 7.

## 3. Results

### 3.1. High FFAs Aggravated Cardiac Microvascular Injury Induced by HG

**Figure 1:**
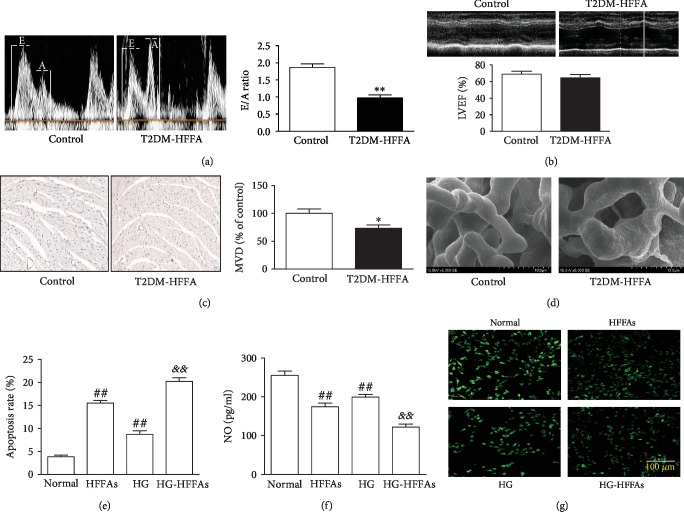
The influence of HG-HFFAs on CMECs. (a) The representative mitral flow patterns detected by cardiac ultrasound (left); the quantification of E/A ratio (right). (b) The percentage of LVEF detected by cardiac ultrasound. (c) The number of cardiac microvessels counted by CD31 immunohistochemical staining (left); the quantification of MVD (right). (d) Cardiac microvascular integrity detected by scanning electron microscope. (e) The apoptosis rate measured by Annexin V-FITC/PI assay kit. (f) NO generation in CMECs measured by ELISA kit. (g) NO production in CMECs detected by staining with DAF-2DA. T2DM-HFFA: type 2 diabetes mellitus with hyper-free fatty acidemia; HG: high glucose; HFFAs: high free fatty acids; HG-HFFAs: high glucose and high free fatty acids; E/A: early mitral diastolic wave/late mitral diastolic wave; LVEF: left ventricular ejection fraction; MVD: microvessel density; ELISA: enzyme-linked immunosorbent assay. Data are expressed as mean ± SEM, *n* = 6‐8/group. ^∗^*P* < 0.05, ^∗∗^*P* < 0.01 vs. control; ^##^*P* < 0.01 vs. normal; ^&&^*P* < 0.01 vs. HG.

We established mouse models of T2DM-HFFA to observe pathological changes in cardiac microvasculature and function. Compared with the control mice, serum levels of glucose, insulin, and FFAs increased significantly in diabetic mice ([Supplementary-material supplementary-material-1]), revealing that we prepared the basic platform for our experiments. Echocardiography showed that E/A ratio, but not LVEF percentage, was decreased obviously in diabetic mice (Figures 1(a) and 1(b)), hinting at that T2DM gives rise to ventricular diastolic dysfunction. Moreover, in diabetic mice, the number of cardiac microvessels decreased remarkably (Figure 1(c)), and those remaining microvessels became rough and uneven (Figure 1(d)). In view of the fact that cardiac microvessel is composed of monolayer endothelial cells, CMECs were isolated from neonatal mice. More than 95% of the cells were positively identified by the endothelial cell marker, vWF and CD31 ([Supplementary-material supplementary-material-1]). HG significantly enhanced CMECs apoptosis and reduced cellular NO content, all of which were further deteriorated by intervention of high FFAs (HFFAs) (Figures 1(e)–1(g)). We also repeated the main experiments on CMECs isolated from the control and T2DM-HFFA mice; the computational data from the T2DM-HFFA modeling largely indicated similar trends with the previous experimental results. T2DM-HFFA not only exacerbated endothelial apoptosis but also further inhibited NO generation ([Supplementary-material supplementary-material-1] and [Supplementary-material supplementary-material-1]). These data suggest that disorder of FFAs metabolism is able to aggravate cardiac microvascular injury induced by HG.

### 3.2. Plin5 Deletion Increased Intracellular FFAs Content in CMECs

**Figure 2:**
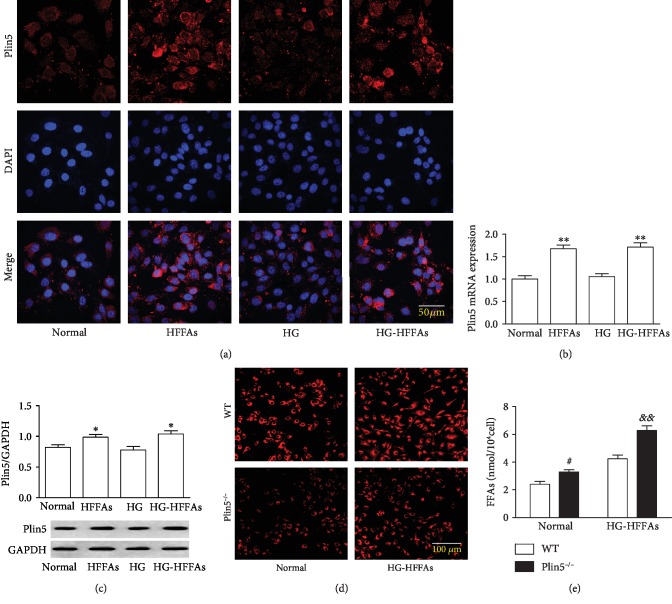
The effects of HG or HFFAs on Plin5 expression in CMECs. (a) Plin5 expression detected by immunofluorescence staining. (b) The mRNA level of Plin5 in CMECs measured by qRT-PCR. (c) The protein level of Plin5 in CMECs determined by western blot. (d) The content of LDs in CMECs detected by Nile red staining. (e) The intracellular level of FFAs in CMECs measured by Free Fatty Acid Quantitation Kit. qRT-PCR: quantitative real-time polymerase chain reaction; HG: high glucose; HFFAs: high free fatty acids; HG-HFFAs: high glucose and high free fatty acids; DAPI: 4′,6-diamidino-2-phenylindole. Data are expressed as mean ± SEM, *n* = 6‐8/group. ^∗^*P* < 0.05 vs. normal; ^∗∗^*P* < 0.01 vs. normal; ^#^*P* < 0.05 vs. WT in normal; ^&&^*P* < 0.01 vs. WT in HG-HFFAs.

In order to clarify the concrete role of Plin5 in metabolism of FFAs, immunofluorescence, qRT-PCR and western blots were introduced to judge Plin5 changes in CMECs. As a result, HFFAs, but not HG, enhanced Plin5 expression (Figures 2(a)–2(c)). In addition, RT-PCR was used to detect genotypes in mice ([Supplementary-material supplementary-material-1]), and CMECs were isolated from the WT and Plin5^−/−^ mouse hearts. Nile red staining showed that HG-HFFAs intervention increased intracellular LDs content, and Plin5 deletion significantly reduced intracellular LDs content (Figure 2(d)), whereas, compared with CMECs in WT, Plin5 deficiency had increased intracellular FFAs content under the condition of normal and HG-HFFAs (Figure 2(e)). These results reveal that Plin5 plays a critical role in the FFAs metabolism of CMECs, which may affect the survival and function of CMECs.

### 3.3. Plin5 Deficiency Exacerbated CMECs Injury Induced by HG-HFFAs

**Figure 3:**
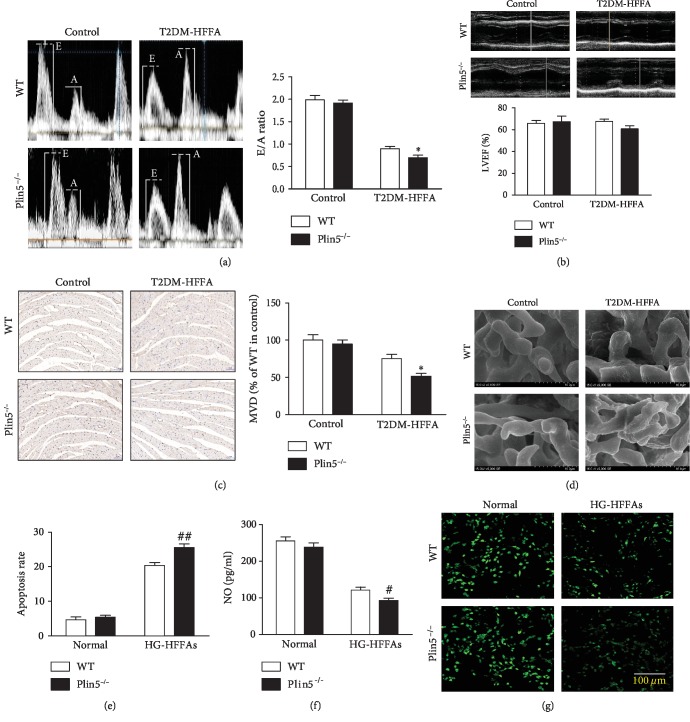
The influence of Plin5 deficiency on CMEC injury induced by HG-HFFAs. (a) The representative mitral flow patterns detected by cardiac ultrasound (left); the quantification of E/A ratio (right). (b) The percentage of LVEF detected by cardiac ultrasound. (c) The number of cardiac microvessels counted by CD31 immunohistochemical staining (left); the quantification of MVD (right). (d) Cardiac microvascular integrity detected by scanning electron microscope. (e) The apoptosis rate measured by Annexin V-FITC/PI assay kit. (f) NO generation in CMECs measured by ELISA kit. (g) NO production in CMECs detected by staining with DAF-2DA. WT: wild type; T2DM-HFFA: type 2 diabetes mellitus with hyper-free fatty acidemia; HG: high glucose; HFFAs: high free fatty acids; HG-HFFAs: high glucose and high free fatty acids; E/A: early mitral diastolic wave/late mitral diastolic wave; LVEF: left ventricular ejection fraction; MVD: microvessel density; ELISA: enzyme-linked immunosorbent assay. Data are expressed as mean ± SEM, *n* = 6‐8/group. ^∗^*P* < 0.05 vs. WT in T2DM-HFFA; ^#^*P* < 0.05, ^##^*P* < 0.01 vs. WT in HG-HFFAs.

Although Plin5 is reported to protect cells from lipid toxicity via preventing excessive FFAs release from TG, its role in lipid metabolism of vascular endothelium has not been investigated. Here, we found that Plin5 knockout had no significant effects on the level of body weight, blood glucose, insulin, and blood lipid ([Supplementary-material supplementary-material-1]). Echocardiographic findings suggested that Plin5 ablation had no significant effect on E/A ratio and LVEF under basic condition. However, it exacerbated ventricular diastolic dysfunction in T2DM-HFFA mice (Figures 3(a) and 3(b)). Corresponding to the ultrasound findings, imaging of scanning electron microscopy and immunohistochemistry showed that Plin5 ablation hardly affected the number and integrity of cardiac microvessels under basic condition, whereas Plin5 deletion resulted in less number of cardiac microvessels and worse endothelial junctions between CMECs under T2DM-HFFA condition (Figures 3(c) and 3(d)). Moreover, cell apoptosis and NO production in WT were close to those in Plin5^−/−^ under normal condition. But, Plin5 knockout in CMECs exacerbated cell apoptosis and NO reduction induced by HG-HFFAs (Figures 3(e)–3(g)). siRNA was used to knock down the expression of Plin5 in WT; results showed that Plin5 knockdown had no significant effect on the apoptotic rate and NO production in CMECs under normal conditions, and yet it aggravated damage to CMECs under HG-HFFAs conditions ([Supplementary-material supplementary-material-1] and [Supplementary-material supplementary-material-1]). In order to explore the mechanism of decreasing NO production, the activity and protein content of endothelial nitric oxide synthase (eNOS) were detected. Under normal conditions, Plin5 knockout had little effect on the activity and protein content of eNOS in CMECs. Nevertheless, under HG-HFFAs conditions, it did reduce the activity and content of eNOS in CMECs ([Supplementary-material supplementary-material-1] and [Supplementary-material supplementary-material-1]). Thus, Plin5 serves as an essential molecule in the survival and function of CMECs when facing to the insult of HG-HFFAs.

### 3.4. Plin5 Phosphorylation Worsened CMECs Injury Induced by HG-HFFAs

**Figure 4:**
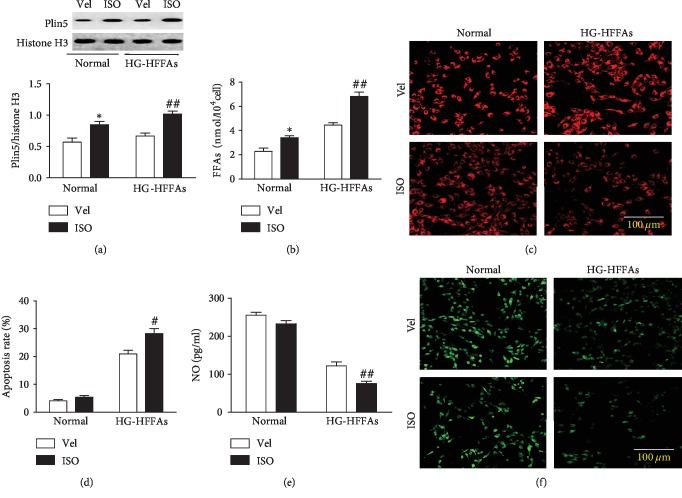
The influence of p-Plin5 on CMECs injury induced by HG-HFFAs. (a) The protein level of Plin5 detected by western blot. (b) The intracellular FFAs content measured by Free Fatty Acid Quantitation Kit. (c) The content of LDs in CMECs determined by Nile red staining. (d) The apoptosis rate. (e) The production of NO measured by ELISA kit. (f) NO generation detected by staining with DAF-2DA in CMECs. Vel: vehicle; ISO: isoproterenol; HG-HFFAs: high glucose and high free fatty acids; ELISA: enzyme-linked immunosorbent assay. Data are expressed as mean ± SEM, *n* = 6‐8/group. ^∗^*P* < 0.05 vs. Vel in normal; ^#^*P* < 0.05, ^##^*P* < 0.01 vs. Vel in HG-HFFAs.

Plin5 phosphorylation also plays an important role in FFAs metabolism. Here, we introduced ISO to activate the phosphorylation of Plin5. With the western blot technology, we found that ISO intervention added endonuclear Plin5 expression in CMECs under normal and HG-HFFAs conditions (Figure 4(a)), which indirectly indicated that cellular Plin5 phosphorylation was enhanced. Meanwhile, after administration of ISO in CMECs, FFAs level significantly increased and LDs content obviously decreased, under normal and HG-HFFAs conditions (Figures 4(b) and 4(c)). Moreover, ISO intervention also deteriorated cell apoptosis and depressed NO generation in CMECs under HG-HFFAs condition. But it is important to note that ISO intervention had no obvious effect on apoptotic rate and NO production under normal condition (Figures 4(d)–4(f)). Further detection found that ISO intervention had little effect on the activity and protein content of eNOS under normal condition; however, it further reduced the activity and content of eNOS in CMECs under HG-HFFAs conditions ([Supplementary-material supplementary-material-1] and [Supplementary-material supplementary-material-1]). In a word, the phosphorylation of Plin5 induced by sympathetic excitement will result in damage to CMECs by enhancing FFAs catabolism.

### 3.5. ROS Overload Induced by Disruption in Plin5 Balance Contributed to CMECs Injury under the Condition of HG-HFFAs

**Figure 5:**
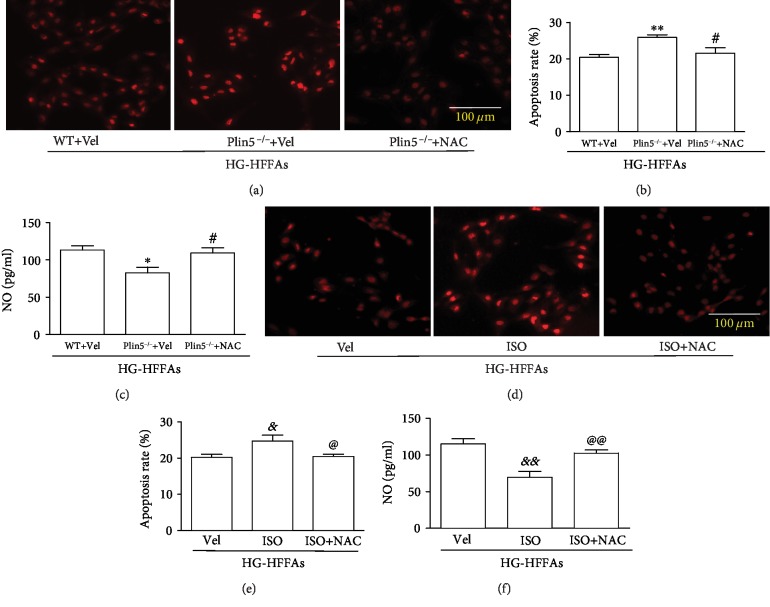
The role of oxidative stress induced by disruption of Plin5 balance in CMECs under the condition of HG-HFFAs. (a, d) ROS in CMECs detected by DHE staining. (b, e) The statistics of apoptosis rate. (c, f) The NO generation in CMECs. ROS: reactive oxygen species; DHE: dihydroethidium; NAC: N-acetyl-cysteine; Vel: vehicle; ISO: isoproterenol; HG-HFFAs: high glucose and high free fatty acid. Data are expressed as mean ± SEM, *n* = 6‐8/group. ^∗^*P* < 0.05, ^∗∗^*P* < 0.01 vs. WT+Vel; ^#^*P* < 0.05 vs. Plin5^−/−^+Vel; ^&^*P* < 0.05, ^&&^*P* < 0.01 vs. Vel; ^@^*P* < 0.05, ^@@^*P* < 0.01 vs. ISO.

It is well known to all that oxidative stress plays a key role in the development of diabetic cardiovascular complications. ROS, as the main activating factor of oxidative stress, is mainly produced by mitochondria. In this study, we explored whether intracellular ROS was affected by the deletion or phosphorylation of Plin5 in the presence of HG-HFFAs. The experimental results showed that both knockout and phosphorylation of Plin5 in CMECs enhanced ROS generation induced by HG-HFFAs (Figures 5(a) and 5(d), [Supplementary-material supplementary-material-1] and [Supplementary-material supplementary-material-1]). Moreover, knockout and phosphorylation of Plin5 enhanced the mRNA expression of CPT-1 ([Supplementary-material supplementary-material-1] and [Supplementary-material supplementary-material-1]). In contrast, NAC, a commonly used intracellular antioxidant, partly reversed CMECs injury induced by Plin5 deletion and phosphorylation under HG-HFFAs condition (Figures 5(b) and 5(c), Figures 5(e) and 5(f)). These results suggest that oxidative stress is the underlying mechanism for CMECs injury induced by Plin5 deletion and phosphorylation.

## 4. Discussion

**Figure 6:**
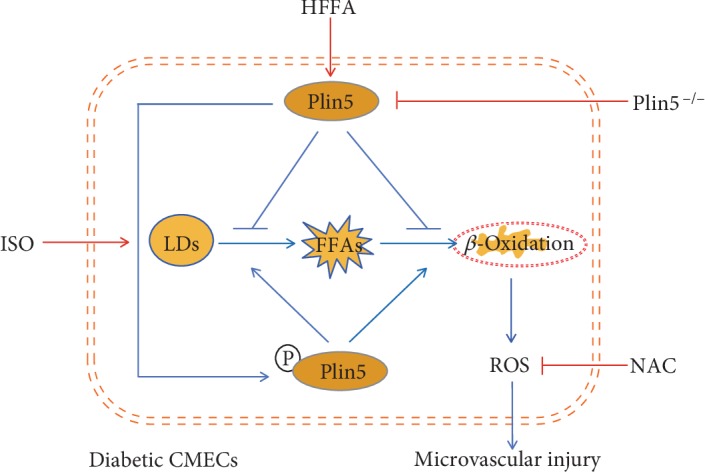
Schematic diagram depicting the bidirectional regulation of Plin5 in the metabolism of intracellular FFAs when facing to T2DM-HFFA. In diabetic CMECs, HFFA, but not hyperglycemia, significantly activates Plin5 expression, and Plin5 is able to inhibit the hydrolysis of LDs to FFAs as well as the mitochondrial *β*-oxidation of FFAs, whereas Plin5 ablation promotes the hydrolysis of LDs to FFAs, enhances the mitochondrial *β*-oxidation of FFAs, gives rise to the excessive generation of mitochondrial ROS, and finally aggravates CMECs injury induced by T2DM-HFFA. On the other hand, the phosphorylation of Plin5 by ISO also promotes the hydrolysis of LDs to FFAs and the mitochondrial *β*-oxidation of FFAs, leading to enhanced oxidative stress and deteriorated CMECs injury. ISO: isoproterenol; LDs: lipid droplets; HFFA: hyper-free fatty acidemia; T2DM-HFFA: type 2 diabetes mellitus with hyper-free fatty acidemia; NAC: N-acetyl-cysteine; ROS: reactive oxygen species; FFAs: free fatty acids.

In this study, we established animal models of T2DM-HFFA by introducing WT and Plin5^−/−^ mice to explore the specific role of Plin5 in CMECs. We finally observed that both deficiency and phosphorylation of Plin5 aggravated CMECs injury induced by T2DM-HFFA via exacerbating oxidative stress. First, we found that HFFAs, but not HG, could obviously activate Plin5 expression in CMECs; in turn, Plin5 ablation was able to increase intracellular content of FFAs in CMECs. Second, we proved that both deficiency and phosphorylation of Plin5 would deteriorate CMECs injury induced by HG-HFFAs. Third, in the absence or phosphorylated mode of Plin5, intracellular ROS overload would be enhanced to cause worse damage to CMECs under the condition of T2DM-HFFA (Figure 6).

It is estimated that there will be 642 million diabetes mellitus by 2040 [41], most of which will suffer from cardiovascular diseases [42]. As an important category of cardiovascular complications in diabetes, microvascular lesions will result in microcirculation disorder and participate in the pathological process of diabetic multiorgan injuries [43]. Gupta et al. have reported that the incidence of microvascular lesions in asymptomatic T2DM patients is 32.55% [44]. As the basic component of cardiac microvessel, CMECs take part in the pathological process of cardiac microvascular angina by regulating the exchange of energy and substance between cardiomyocyte and blood [45, 46]. In addition, CMECs are the earliest and most vulnerable target of diabetic damage to the heart [47]. According to an epidemiological survey in China, 67.1% of T2DM patients are associated with dyslipidemia. In these T2DM patients, HFFA is one of the most common dyslipidemias that is characterized by excessive FFAs in circulation, which can enhance the inflammatory reaction and oxidative stress in endothelial cells, further induce endothelial apoptosis, and participate in insulin resistance and vascular disease [48–51]. Correction of dyslipidemia can significantly reduce the incidence of cardiovascular complications in T2DM. However, up to now, it is rarely reported about CMECs injury in T2DM-HFFA. In our present study, serum TG, CHOL, INS, and FFAs of T2DM-HFFA mice were significantly increased. Then, our study showed that HFFA reduced the number of microvessels in T2DM-HFFA mice and impaired the integrity of microvessels, which might contribute to the dysfunction of cardiac microvascular barrier and lead to myocardial metabolic disorders. Moreover, we found that left ventricular diastolic function was impaired in T2DM-HFFA mice, which might may be related to the disturbance of myocardial energy supply caused by microvascular injury. Furthermore, we also found that HFFAs aggravated cell apoptosis and NO reduction induced by HG in cultured CMECs. In line with our results, Pilz et al. claimed that HFFA increased the risk of ischemic heart disease and the mortality of coronary heart disease [52, 53], and Guo et al. defined HFFA as an independent risk factor for hypertension [54]. Some studies have reported that hyperglycemia and HFFAs induce vascular endothelial cell apoptosis and reduce the synthesis and bioavailability of NO [55–57]. Thus, it is necessary to investigate the specific mechanism of CMECs injury under the condition of HG-HFFAs and explore new targets for clinical intervention.

As mainly formed by central neutral lipids and peripheral phospholipid monolayer, LDs are key regulators for lipid metabolism both in adipose tissues and nonadipose tissues [58]. As an important member of the PAT protein family on the surface of LDs, Plin5 has aroused the enthusiasm of many researchers. Plin5 can inhibit the hydrolysis of neutral lipid in LDs through interaction with lipolytic hydrolase such as hormone-sensitive triglyceride lipase (HSL), adipose triglyceride lipase (ATGL), and comparative gene identification-58 (CGI-58), thus preventing excessive generation and oxidation of FFAs [21, 59]. In our experiments, we simulated T2DM-HFFA state in vitro by giving intervention of glucose and FFAs to cultured CMECs. As last, we found that Plin5 was expressed in CMECs derived from mice. The intervention of HFFAs, but not HG, obviously increased the expression of Plin5 at the mRNA and protein levels. Consistently, many studies proved that exogenous intervention of FFAs stimulated Plin5 expression in many kinds of cultured cells [60–62], and the increase in Plin5 may be generally attributed to the activation of peroxisome proliferator-activated receptors (PPARs) [60, 63]. Some literature even claimed that high-fat diet was able to increase Plin5 expression in the liver and skeletal muscles [16, 64, 65]. It was found that Plin5 knockout could increase the content of FFAs in liver tissue of mice [16]. In our current studies of CMECs, we found that Plin5 ablation could reduce the content of LDs and increase the content of FFAs, suggesting that Plin5 plays an important role in the metabolism of LDs and FFAs in CMECs. Moreover, Plin5 knockout reduced the number of cardiac microvessels and aggravated the injury of microvascular integrity in T2DM-HFFA mice. In addition, Plin5 knockout aggravated cardiac diastolic dysfunction, but had little effects on cardiac systolic function in T2DM-HFFA mice. So, we speculate that the effects of Plin5 deletion on the number and structure of cardiac microvasculature contribute to the deterioration of diastolic dysfunction. The inconformity in changes of cardiac function may be due to differences in the severity of cardiac microvascular and myocardial damage. Consistent with our data, other studies have shown that Plin5 knockout is considered to have no effect on cardiac function in the physiological state [31, 66], but aggravate cardiac dysfunction induced by old age or ischemic stimuli in mice [14]. Furthermore, we found that Plin5 deficiency increased apoptosis rate and decreased NO synthesis in CMECs under the condition of HG-HFFAs. It is well known that eNOS plays a decisive role in the production and utilization of NO in endothelial cells. Studies have confirmed that FFAs can inhibit eNOS mRNA expression and regulate eNOS activity by increasing oxidative stress and inflammatory burden in aortic endothelial cells of rats [56]. Our study also demonstrated that Plin5 deletion reduced eNOS content in CMECs under HG-HFFAs condition, which may contributed to the reduction of NO generation. Similarly, Ibrahim et al. proved that in patients of obesity, diabetes and nonalcoholic fatty liver, the core of lipid toxicity was the excessive decomposition of TG to FFAs and the accumulation of FFAs [67]. Therefore, Plin5 participates in the metabolism of FFAs in CMECs and its deficiency may aggravate cardiac microvascular injury induced by T2DM-HFFA. Plin5 can be used as a new indicator to predict the occurrence and development of coronary heart disease.

Interestingly, Plin5 overexpression in the heart caused severe cardiac steatosis and left ventricular hypertrophy [19, 20]. Further study found that Plin5 overexpression did not continue to inhibit LDs decomposition in myocardium, which might be associated with p-Plin5-stimulated hydrolysis of LDs under various stressful conditions [22]. Researches in various aspects showed that PAT protein family members participated in cell lipid metabolism via two states which contained nonphosphorylation and phosphorylation. Under basic conditions, Plin5 was bound to ATGL and CGI-58, respectively, to prevent the combination of this two, thus inhibiting the hydrolysis of LDs. Plin5 could also promote FFAs enter into LDs to synthesize neutral fat, thus preventing the excessive oxidation of FFAs [21]. On the other hand, adrenaline, secreted by sympathetic excitability in the case of cold, tension, fear, and so on, is able to activate PKA and increase the phosphorylation of Plin5, which may prompt the incidence of cardiovascular disease, especially in T2DM patients. When adrenergic receptors were activated, Plin5 was phosphorylated by PKA activation and promoted the hydrolysis of LDs. Catecholamine stimulation could increase the lipolysis rate by 50 times in adipose tissues [68]. In addition, p-Plin5 also participated in the transfer of FFAs to mitochondrial for *β*-oxidation [13, 22]. The released FFAs from LDs could also increase the transcriptional of PPARs which stimulate gene transcription related to the synthesis and oxidation of mitochondria [21, 24]. At present, there is no specific antibody to detect the phosphorylation of Plin5, but it has been reported that only p-Plin5 can enter into the nucleus to play a corresponding role [24]. Our studies revealed that, in WT group, ISO stimulation increased Plin5 expression in the nucleus. More importantly, ISO enhanced the hydrolysis of LDs, raised the intracellular level of FFAs, reduced intracellular eNOS level, and exacerbated CMECs injury induced by HG-HFFAs. It should be noted that although ISO stimulation increased intracellular FFAs levels in basic condition, it did not cause damage to CMECs which may be related to cellular self-regulation. These results indicated that ISO intervention was able to increase the phosphorylation of Plin5 in CMECs and aggravate CMECs injury induced by HG-HFFAs. In other words, these results suggested that the sympathetic excitement in T2DM-HFFA patients may give rise to functional and structural damage to the cardiac microvasculature, finally leading to sudden cardiac death. Further study of these findings may help uncover the pathological mechanism why strenuous exercise can give rise to angina or even sudden cardiac death.

However, in a study of rats, it was found that the degree in phosphorylation of Plin5 was not consistent with the hydrolysis of LDs in skeletal muscle cells [23]. Interestingly, Kuramoto et al. also proved that in fed condition, Plin5^−/−^ reduced the content of FFAs in the liver, but not in the heart and soleus muscle, whereas in fasted condition, Plin5^−/−^ increased FFAs content in the liver and reduced it in the heart and soleus muscle. This discrepancy may prove again that the concrete roles of Plin5 depend on the species, the tissues, and eating or not.

The generation and removal of free radicals in the body are in equilibrium under normal conditions [69]. But in the pathological condition, the production of free radicals is increased, and the ability of antioxidation in body is decreased, resulting in lipid peroxidation, protein degeneration, and DNA damage [70]. The main marker of oxidative stress is excessive ROS, which is mainly produced from mitochondria. Studies have shown that endothelial cells supply their own energy mainly through glycolysis [71], but it has been reported that endothelial cells still can carry out large amounts of mitochondrial *β*-oxidation for energy supply under the stimulation of acute HFFAs [17]. Thus, we believe that mitochondrial ROS produced from *β*-oxidation cannot be ignored in the CMECs injury of T2DM-HFFA. Consistently, our studies revealed again that increased intracellular FFAs content led to excessive production of ROS. As well known, CPT-1 is the first speed-limiting enzyme in the process of mitochondrial fatty acid oxidation, and it can catalyze the long-chain fatty acids from the cytoplasm into the mitochondria [72, 73]. In in vitro cell experiment, we found that the deletion and phosphorylation of Plin5 in CMECs increased mRNA expression of CPT-1 and raised intracellular level of ROS under the condition of HG-HFFAs. Consistently, de Barros Reis et al. reported that the increase in CPT-1 activity enhanced FFAs oxidation in islet cells [74]. Moreover, NAC intervention reduced the content of ROS, partly reversed the CMECs injury induced by the deletion and phosphorylation of Plin5. In line with our studies, many previous studies claimed that Plin5 knockout was able to increase the level of FFAs, enhance the *β*-oxidation of FFAs, and ultimately increase the production of ROS [14, 31]. In myocardial ischemia-reperfusion injury, it has been reported that Plin5 ablation can increase the content of ROS and malondialdehyde (MDA), but reduce the level of superoxide dismutase (SOD) [31]. Therefore, these results suggest that ROS overload derived from FFAs *β*-oxidation contributes to CMECs injury induced by the disruption of Plin5 balance in T2DM-HFFA.

## 5. Conclusion

HFFAs can aggravate HG-induced cardiac microvascular injury; Plin5 in CMECs plays a bidirectionally role in FFAs metabolism via the states of nonphosphorylation and phosphorylation; the deletion or the phosphorylation of Plin5 will break the balance of FFAs metabolism, result in the excessive production of FFAs and the overload of ROS, and eventually aggravate the CMECs injury induced by T2DM-HFFA. This suggests that only appropriate expression and phosphorylation of Plin5 are beneficial to energy supply and fine regulation of FFAs metabolism in CMECs. In short, our study suggests that Plin5 can guard the orderly metabolism of FFAs in CMECs in different states, and it can be explored as a new target for the prevention and treatment of microvascular complications in T2DM-HFFA.

## Data Availability

The data used to support the findings of this study are included within the article.

## Abbreviations

HFFA: Hyper-free fatty academia
T2DM: Type 2 diabetes mellitus
Plin5: Perilipin 5
FFAs: Free fatty acids
CMECs: Cardiac microvascular endothelial cells
HG: High glucose
HFFAs: High FFAs
LDs: Lipid droplets
NO: Nitric oxide
ROS: Reactive oxygen species
eNOS: Endothelial nitric oxide synthase
NEFAs: Nonesterified fatty acids
CMD: Coronary microvascular diseases
TG: Triglyceride
PKA: Protein kinase A
PGC-1*α*: Peroxisome proliferator-activated receptor gamma coactivator 1-*α*
RNS: Reactive nitrogen species
STZ: Streptozocin
CHOL: Cholesterol
INS: Insulin
ABS: Acrylonitrile butadiene styrene
BSA: Bovine serum albumin
LVEF: Left ventricular ejection fraction
E/A: Left ventricular early mitral diastolic wave/late mitral diastolic wave
PBS: Phosphate-buffered saline
FBS: Fetal bovine serum
vWF: von Willebrand factor
ISO: Isoprenaline
LSCM: Laser confocal microscopy
ELISA: Enzyme-linked immunosorbent assay
HBSS: Hank's balanced salt solution
qRT-PCR: Quantitative real-time polymerase chain reaction.

## Supplementary Materials

Supplementary Materials Table 1: basal physiological parameters in all kinds of mice. Plin5: perilipin 5; BW: body weight; HW: heart weight; GLU: glucose; INS: insulin; TG: triglyceride; FFAs: free fatty acids; CHOL: total cholesterol. Presented values are mean ± SEM, *n* = 8‐10/group. ^∗^*P* < 0.05, ^∗∗^*P* < 0.01 vs. WT of the same genotypes. Supplementary Figure 1: identification of cardiac microvascular endothelial cells (CMECs) and T2DM-HFFA caused damage to CMECs. (A) Cell immunofluorescence staining. DAPI: 4′,6-diamidino-2-phenylindole (blue fluorescence); CD31: (green fluorescence); vWF: von Willebrand factor (red fluorescence). (B) The apoptosis rate measured by Annexin V-FITC/PI assay kit. (C) NO generation in CMECs measured by ELISA kit. T2DM-HFFA: type 2 diabetes mellitus with hyper-free fatty acidemia; ELISA: enzyme-linked immunosorbent assay. Data are expressed as mean ± SEM, *n* = 6‐8/group. ^*θ*^*P* < 0.05, ^*θθ*^*P* < 0.01 vs. control. Supplementary Figure 2: identification of mouse genotypes. Polymerase chain reaction was used to identify WT and Plin5^−/−^ mice. Supplementary Figure 3: the impact of Plin5 knockdown on the injury of CMECs induced by HG-HFFAs. (A) The apoptosis rate measured by Annexin V-FITC/PI assay kit. (B) NO generation in CMECs measured by ELISA kit. HG-HFFAs, high glucose and high free fatty acids; Scra siRNA, scrambled siRNA; ELISA, enzyme-linked immunosorbent assay. Data are expressed as mean ± SEM, *n* = 6‐8/group. ^∗^*P* < 0.05 vs. HG-HFFAs of Scra siRNA. Supplementary Figure 4: the effect of Plin5 knockout on eNOS in CMECs. (A) The activity of eNOS measured by eNOS Quantitation Kit. (B) The protein level of eNOS in CMECs determined by western blot. WT, wild type; eNOS, endothelial nitric oxide synthase; HG-HFFAs, high glucose and high free fatty acids. Data are expressed as mean ± SEM, *n* = 6‐8/group. ^∗∗^*P* < 0.01 vs. WT of normal; ^##^*P* < 0.01 vs. Plin5^−/−^ of normal; ^&^*P* < 0.05 vs. WT of HG-HFFAs. Supplementary Figure 5: the effect of Plin5 phosphorylation on eNOS in CMECs. (A) The activity of eNOS measured by eNOS Quantitation Kit. (B) The protein level of eNOS in CMECs determined by western blot. Vel, vehicle; ISO, isoproterenol; eNOS, endothelial nitric oxide synthase; HG-HFFAs, high glucose and high free fatty acids. Data are expressed as mean ± SEM, *n* = 6‐8/group. ^∗∗^*P* < 0.01 vs. Vel of normal; ^##^*P* < 0.01 vs. ISO of normal; ^&^*P* < 0.05 vs. Vel of HG-HFFAs. Supplementary Figure 6: the effect of Plin5/p-Plin5 on the mRNA expression of CPT-1 and ROS content in CMECs under the condition of HG-HFFAs. (A, B) The production of ROS in CMECs was measured by ELISA kit. (C, D) CPT-1 mRNA expression in CMECs measured by qRT-PCR. CPT-1, carnitine palmitoyltransferase I; HG-HFFAs, high glucose and high free fatty acids; NAC, N-acetyl-cysteine; Vel, vehicle; ISO, isoproterenol; ELISA, enzyme-linked immunosorbent assay; ROS, reactive oxygen species; qRT-PCR, quantitative real-time polymerase chain reaction. Presented values are mean ± SEM, *n* = 6‐8/group. ^∗∗^*P* < 0.01 vs. WT+Vel; ^##^*P* < 0.01 vs. Plin5^−/−^+Vel; ^&&^*P* < 0.01 vs. vehicle; ^@@^*P* < 0.01 vs. ISO.
