# Supplementary material for: Plin5/p-Plin5 Guards Diabetic CMECs by Regulating FFAs Metabolism Bidirectionally
Source: Oxid Med Cell Longev. 2019 Oct 17;2019:8690746. doi: 10.1155/2019/8690746 (PMC6854993; doi:10.1155/2019/8690746)

**Supplemental Tables**

**Table 1 Basal physiological parameters in all kinds of mice**


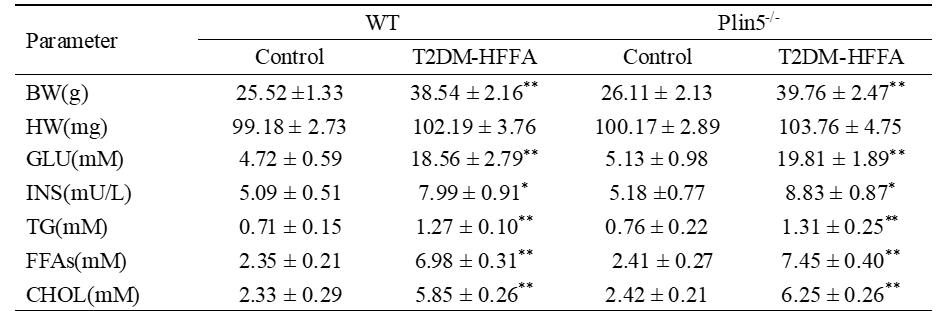


**Note:** Plin5, [perilipin 5](http://www.baidu.com/link?url=Zk9qveiKHauqQurcAhirYueV_AI3y_uwDCREVR1hUU11YIpDYq7jOkV3bsq6YID1SYiTq1dnFTMRwI3JfmMP6K); BW, Body weight; HW, Heart weight; GLU, Glucose; INS, Insulin; TG, Triglyceride; FFAs, Free fatty acids; CHOL, total cholesterol. Presented values are means±SEM, n=8-10/group. *^*^P<0.05*, *^**^P<0.01* vs. WT of the same genotypes.

**Supplementary Figure Legends**

**Supplementary Figure 1. Identification of cardiac microvascular endothelial cells (****CMECs) and T2DM-HFFA caused damage to CMECs.**

**(A)** Cell immunofluorescence staining. DAPI, 4’,6-diamidino-2-phenylindole (blue fluorescence); CD31 (green fluorescence); vWF, von Willebrand factor (red fluorescence). **(B)** The apoptosis rate measured by Annexin V-FITC/PI assay kit. **(C)** NO generation in CMECs measured by ELISA kit. T2DM-HFFA, type 2 diabetes mellitus with hyper-free fatty acidemia; ELISA, enzyme-linked immunosorbent assay. Data are expressed as mean±SEM, n=6-8/group. *^θ^P*<0.05, *^θθ^P*<0.01 vs. Control.


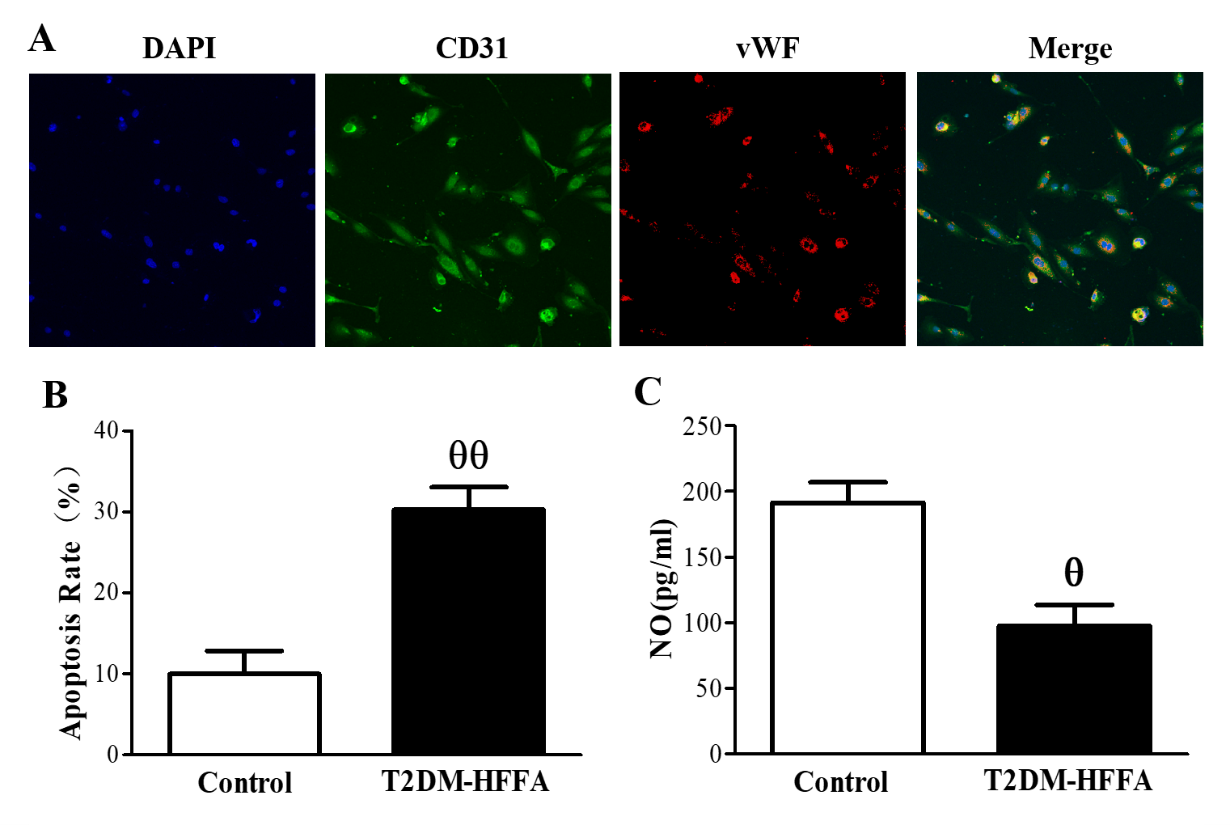


**Supplementary Figure 2. Identification of mouse genotypes.**

Polymerase chain reaction was used to identify WT and Plin5^−/−^ mice.


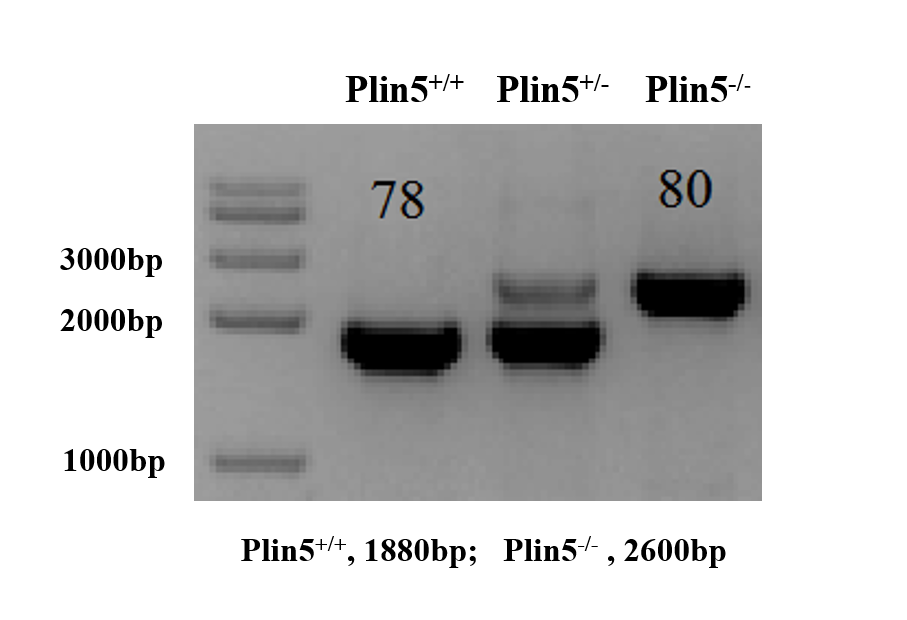


**Supplementary Figure 3. Impact of Plin5 knockdown on the injury of CMECs induced by HG-HFFAs.**

**(A)** The apoptosis rate measured by Annexin V-FITC/PI assay kit. **(B)** NO generation in CMECs measured by ELISA kit. HG-HFFAs, high glucose and high free fatty acids; Scra siRNA, scrambled siRNA; ELISA, enzyme-linked immunosorbent assay. Data are expressed as mean±SEM, n=6-8/group. **P*<0.05 vs. HG-HFFAs of Scra siRNA.


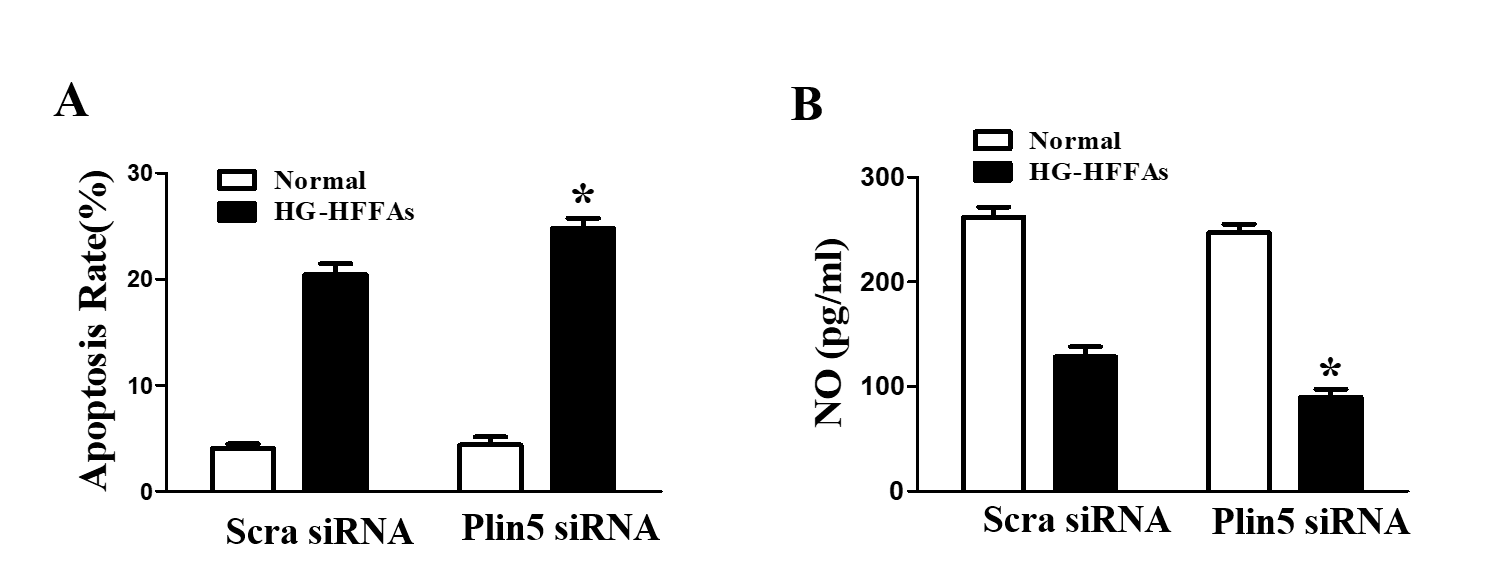


**Supplementary Figure 4. Effect of Plin5 knockout on eNOS in CMECs.**

**(A)** The activity of eNOS measured by eNOS Quantitation Kit. **(B)** The protein level of eNOS in CMECs determined by western blot. WT, wide type; eNOS, endothelial nitric oxide synthase; HG-HFFAs, high glucose and high free fatty acids. Data are expressed as mean±SEM, n=6-8/group. ***P*<0.01 vs. WT of Normal; ^##^*P*<0.01 vs. Plin5^-/-^ of Normal; ^&^*P*<0.05 vs. WT of HG-HFFAs.


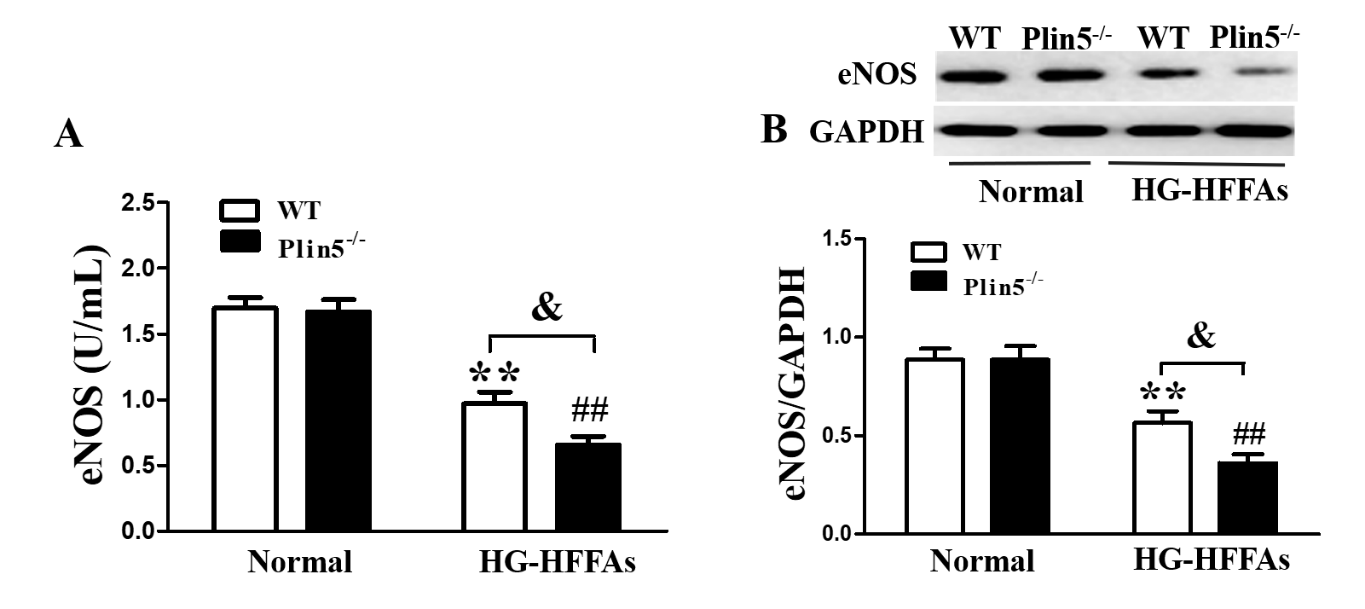


**Supplementary Figure 5. Effect of phosphorylation of Plin5 on eNOS in CMECs.**

1. The activity of eNOS measured by eNOS Quantitation Kit. **(B)** The protein level of eNOS in CMECs determined by western blot. Vel, vehicle; ISO, isoproterenol; eNOS, endothelial nitric oxide synthase; HG-HFFAs, high glucose and high free fatty acids. Data are expressed as mean±SEM, n=6-8/group. ***P*<0.01 vs. Vel of Normal; ^##^*P*<0.01 vs. ISO of Normal; ^&^*P*<0.05 vs. Vel of HG-HFFAs.


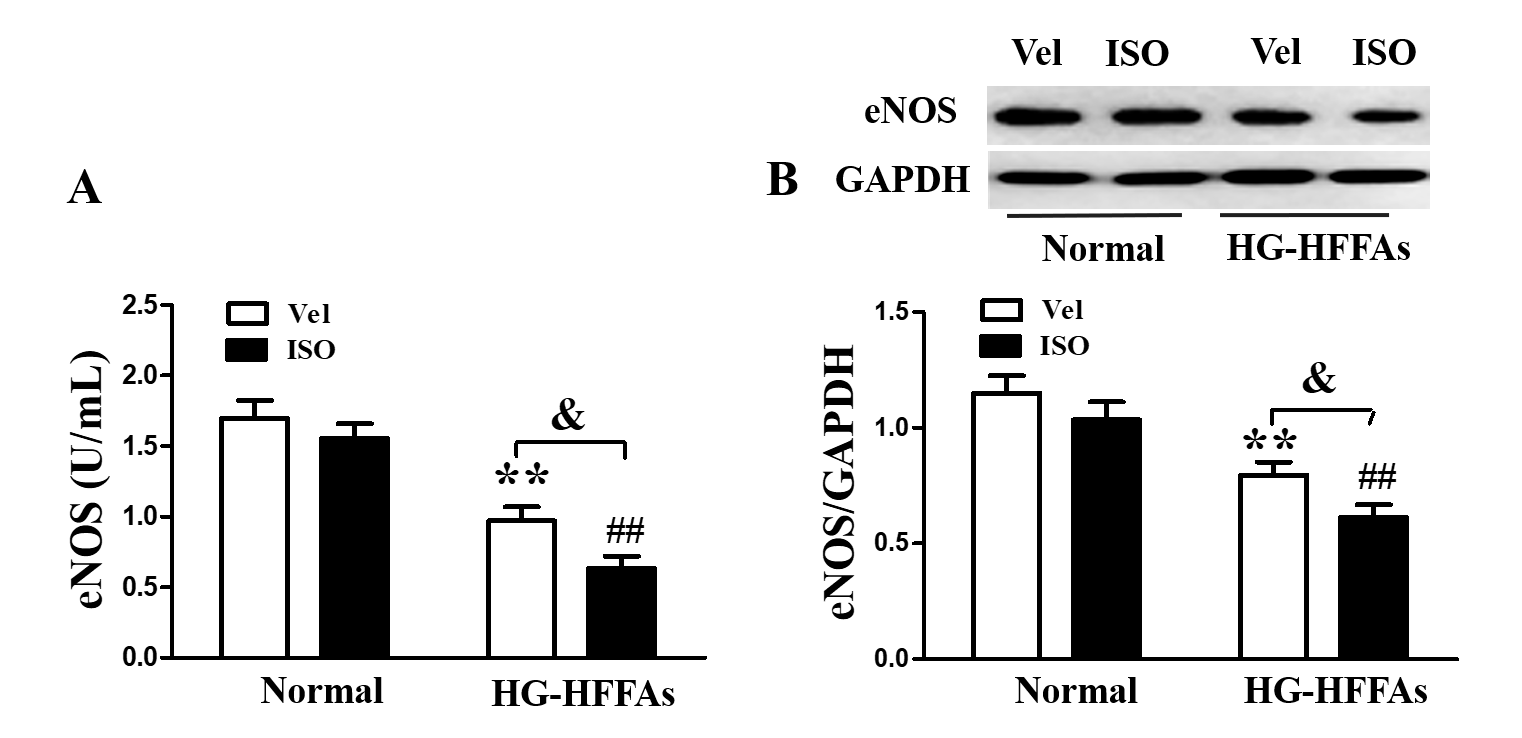


**Supplementary Figure 5. Effect of Plin5/p-Plin5 on the mRNA expression of CPT-1 and ROS content in CMECs under the condition of HG-HFFAs.**

**(A, B)** The production of NO in CMECs was measured by ELISA Kit; **(C, D)** CPT-1 mRNA expression in CMECs measured by qRT-PCR. CPT-1, Carnitine palmitoyltransferase I; HG-HFFAs, high glucose and high free fatty acids; NAC, N-acetyl-cysteine; Vel, vehicle; ISO, isoproterenol; ELISA, enzyme-linked immunosorbent assay; ROS, reactive oxygen species; qRT-PCR, quantitative real time polymerase chain reaction. Presented values are mean±SEM, n=6-8/group. ^**^*P*<0.01 vs. WT+Vel; ^##^*P*<0.01 vs. Plin5^-/-^ +Vel; ^&&^*P*<0.01 vs. Vehicle; ^@@^*P*<0.01 vs. ISO.


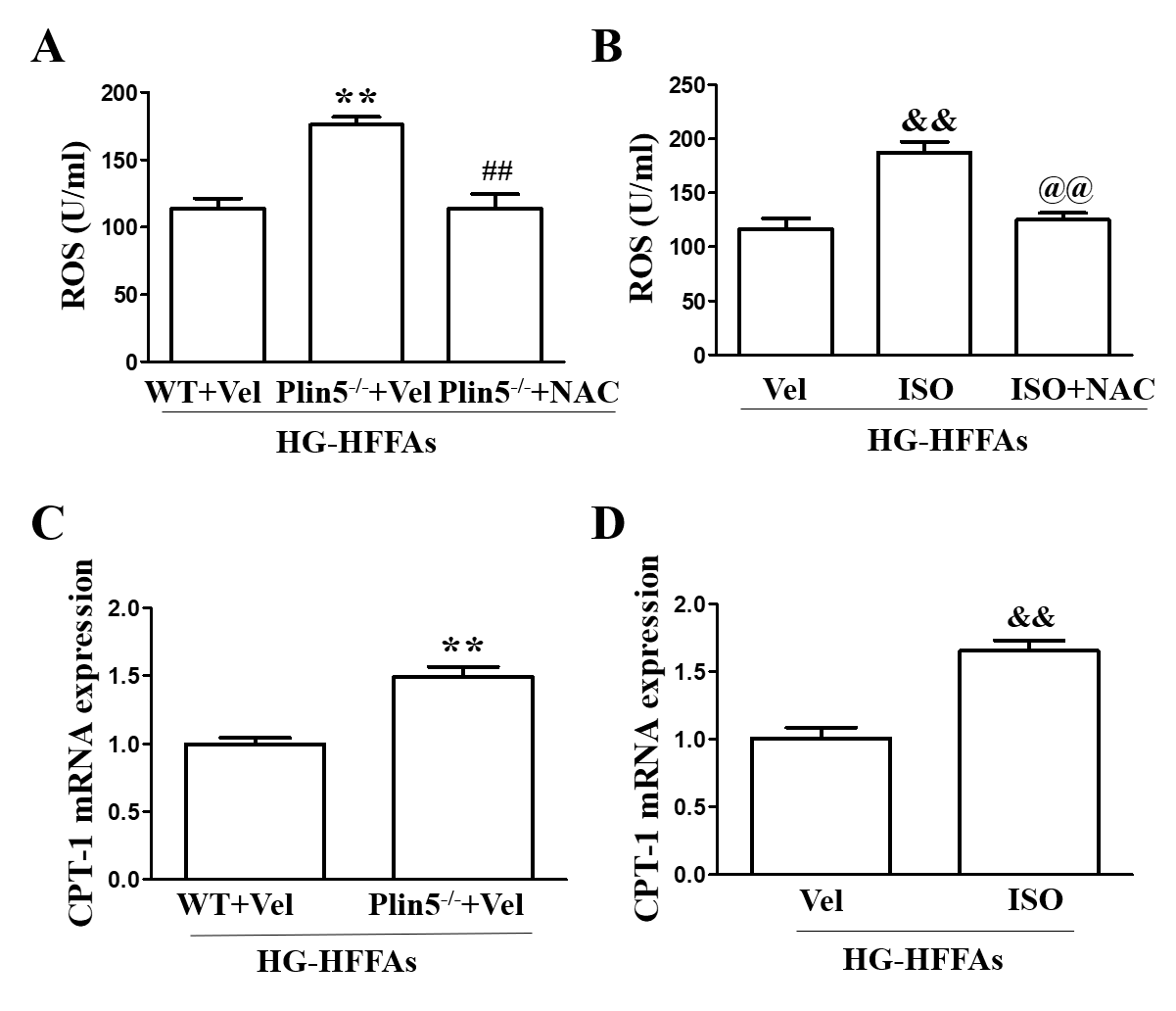

Supplement: Supplementary Materials — Table 1: basal physiological parameters in all kinds of mice. Plin5: perilipin 5; BW: body weight; HW: heart weight; GLU: glucose; INS: insulin; TG: triglyceride; FFAs: free fatty acids; CHOL: total cholesterol. Presented values are mean ± SEM, n = 8‐10/group. ∗P < 0.05, ∗∗P < 0.01 vs. WT of the same genotypes. Supplementary Figure 1: identification of cardiac microvascular endothelial cells (CMECs) and T2DM-HFFA caused damage to CMECs. (A) Cell immunofluorescence staining. DAPI: 4′,6-diamidino-2-phenylindole (blue fluorescence); CD31: (green fluorescence); vWF: von Willebrand factor (red fluorescence). (B) The apoptosis rate measured by Annexin V-FITC/PI assay kit. (C) NO generation in CMECs measured by ELISA kit. T2DM-HFFA: type 2 diabetes mellitus with hyper-free fatty acidemia; ELISA: enzyme-linked immunosorbent assay. Data are expressed as mean ± SEM, n = 6‐8/group. θP < 0.05, θθP < 0.01 vs. control. Supplementary Figure 2: identification of mouse genotypes. Polymerase chain reaction was used to identify WT and Plin5−/− mice. Supplementary Figure 3: the impact of Plin5 knockdown on the injury of CMECs induced by HG-HFFAs. (A) The apoptosis rate measured by Annexin V-FITC/PI assay kit. (B) NO generation in CMECs measured by ELISA kit. HG-HFFAs, high glucose and high free fatty acids; Scra siRNA, scrambled siRNA; ELISA, enzyme-linked immunosorbent assay. Data are expressed as mean ± SEM, n = 6‐8/group. ∗P < 0.05 vs. HG-HFFAs of Scra siRNA. Supplementary Figure 4: the effect of Plin5 knockout on eNOS in CMECs. (A) The activity of eNOS measured by eNOS Quantitation Kit. (B) The protein level of eNOS in CMECs determined by western blot. WT, wild type; eNOS, endothelial nitric oxide synthase; HG-HFFAs, high glucose and high free fatty acids. Data are expressed as mean ± SEM, n = 6‐8/group. ∗∗P < 0.01 vs. WT of normal; ##P < 0.01 vs. Plin5−/− of normal; &P < 0.05 vs. WT of HG-HFFAs. Supplementary Figure 5: the effect of Plin5 phosphorylation on eNOS in CMECs. (A) The [file 8690746.f1.docx]
